# Machine-learning model for predicting oliguria in critically ill patients

**DOI:** 10.1038/s41598-024-51476-y

**Published:** 2024-01-11

**Authors:** Yasuo Yamao, Takehiko Oami, Jun Yamabe, Nozomi Takahashi, Taka-aki Nakada

**Affiliations:** 1 Department of Emergency and Critical Care Medicine, Chiba University Graduate School of Medicine, 1-8-1 Inohana, Chuo, Chiba, 260-8677 Japan; 2 Smart119 Inc, Chiba, Japan

**Keywords:** Computational biology and bioinformatics, Nephrology, Diagnosis

## Abstract

This retrospective cohort study aimed to develop and evaluate a machine-learning algorithm for predicting oliguria, a sign of acute kidney injury (AKI). To this end, electronic health record data from consecutive patients admitted to the intensive care unit (ICU) between 2010 and 2019 were used and oliguria was defined as a urine output of less than 0.5 mL/kg/h. Furthermore, a light-gradient boosting machine was used for model development. Among the 9,241 patients who participated in the study, the proportions of patients with urine output < 0.5 mL/kg/h for 6 h and with AKI during the ICU stay were 27.4% and 30.2%, respectively. The area under the curve (AUC) values provided by the prediction algorithm for the onset of oliguria at 6 h and 72 h using 28 clinically relevant variables were 0.964 (a 95% confidence interval (CI) of 0.963–0.965) and 0.916 (a 95% CI of 0.914–0.918), respectively. The Shapley additive explanation analysis for predicting oliguria at 6 h identified urine values, severity scores, serum creatinine, oxygen partial pressure, fibrinogen/fibrin degradation products, interleukin-6, and peripheral temperature as important variables. Thus, this study demonstrates that a machine-learning algorithm can accurately predict oliguria onset in ICU patients, suggesting the importance of oliguria in the early diagnosis and optimal management of AKI.

## Introduction

Acute kidney injury (AKI), which is defined as a rapid increase in serum creatinine or decrease in urine output, is one of the leading causes of complications during intensive care unit (ICU) admission, resulting in persistent organ dysfunction and increased mortality^1–4^. Although early detection of AKI and prompt intervention can improve the prognosis of critically ill patients, none of the procedures, including close monitoring of vital signs, blood tests, and urine analysis, provides promising solutions. Serum creatinine, which is used to diagnose AKI, has a reduced reliability for the early detection of the pathology^5–9^.

A recent study showed that oliguria, which is generally defined as a urine output of less than 0.5 mL/kg/h over 6 h, was associated with 90-day mortality irrespective of elevations in serum creatinine levels, exhibiting important diagnostic implications for oliguria in AKI management^10^. In addition, a prospective observational study showed that continuous monitoring of urine output could identify more patients with AKI earlier than serum creatinine alone^11^. Therefore, management of AKI with accurate prediction of oliguria may be a promising strategy to understand the complex pathophysiology of critical illnesses better.

With the advancement of artificial intelligence, a substantial number of prediction models using machine-learning algorithms have demonstrated high accuracy in predicting mortality and clinical outcomes in ICU patients, including the detection of AKI^12–14^. In terms of oliguria, a previous study reported a machine-learning approach for predicting urine output in patients with sepsis after fluid administration^15^. However, the accuracy of a machine-learning model in predicting oliguria in a general ICU setting remains underexplored.

Therefore, we hypothesized that a machine-learning model could predict the onset of oliguria in patients admitted to an ICU. This study aimed to develop a machine-learning algorithm for predicting oliguria in patients at 6 and 72 h, from an arbitrary period, during their ICU stay and to evaluate the accuracy of the developed algorithm using a large database from a single-center surgical/medical mixed ICU.

## Results

### Characteristics of the cohort

Of the 14,105 patients screened, 4,745 patients without documented body weight and 119 patients on maintenance dialysis were excluded; therefore, only 9,241 patients were included in the study (Supplementary File: Fig. S1). No significant differences were observed between the training and test data. In the entire cohort, the proportions of patients with urine output < 0.5 mL/kg/h for 6 h and with AKI during their ICU stay were 27.4% and 30.2%, respectively (Table 1).

**Table 1:** Patient characteristics and outcomes in the training and test cohorts.

Variables	Training cohort (n = 7392)	Test cohort (n = 1849)
Demographic data		
Age, years	67 (56–75)	67 (55–75)
Male, n (%)	4,599 (62.2)	1,106 (59.8)
Body mass index	22.0 (19.2–24.9)	22.1 (19.1–24.8)
Sepsis/septic shock	264 (3.6)	75 (4.1)
Heart failure	276 (3.7)	75 (4.1)
Department		
Surgery	3,448 (46.6)	853 (46.1)
Internal medicine	1,193 (16.1)	299 (16.2)
Emergency medicine	444 (6.0)	133 (7.2)
Others	2,307 (31.2)	564 (30.5)
Missing values	6 (0.1)	2 (0.1)
Admission route		
Emergency room	484 (6.5)	126 (6.8)
General ward	544 (7.4)	135 (7.3)
Operating room, emergency	175 (2.4)	46 (2.4)
Operating room, elective	2,560 (34.6)	615 (33.3)
Other hospital	174 (2.4)	56 (3.0)
Missing values	3436 (46.5)	868 (46.9)
Acute kidney injury during ICU stay, n (%)	2,243 (30.3)	544 (29.4)
Physiological data^a^		
Urine output (mL/kg/h)	0.9 (0.4–1.7)	0.9 (0.4–1.8)
Urine output < 0.5 mL/kg/h for 6 h, n (%)	2,028 (27.4)	501 (27.1)
Fluid balance (mL/day)	− 742.0 (− 1,538.3 to − 190.0)	− 745.0 (− 1,577.1 to − 161.0)
Laboratory data on ICU admission		
Serum creatinine (mg/dL)	0.8 (0.6–1.1)	0.8 (0.6–1.2)
White blood cell (× 10^3^/mm^3^)	10.3 (7.7–13.6)	10.1 (7.6–13.2)
Interleukin-6 (pg/mL)	98.2 (40.6–257.3)	98.7 (35.3–233.8)
Fibrinogen degradation products (μg/mL)	14.5 (7.7–32.5)	13.0 (7.0–25.3)
Lactate (mmol/L)	1.2 (0.9–1.9)	1.2 (0.8–1.9)
APACHE II score	19 (14–27)	20 (14–28)
SOFA score	5 (2–7)	5 (3–8)
Therapeutic interventions, n (%)		
Cardiovascular agents	2,096 (28.4)	557 (30.1)
Diuretics	874 (11.8)	228 (12.3)
Renal replacement therapy, n (%)	674 (9.1)	182 (9.8)
Transfusion	1,889 (25.6)	472 (25.5)
Outcomes		
Length of ICU stay, days	1 (1–3)	1 (1–3)
Survival on ICU discharge, n (%)	7,161 (96.9)	1,762 (95.3)

Data are expressed as median (interquartile range) for continuous variables and as numbers (%) for categorical variables

*APACHE* acute physiology and chronic health evaluation; *SOFA* sequential organ failure assessment; *ICU* intensive care unit

^a^The average data during the ICU stay

### Selection of variables

**Figure 1:**
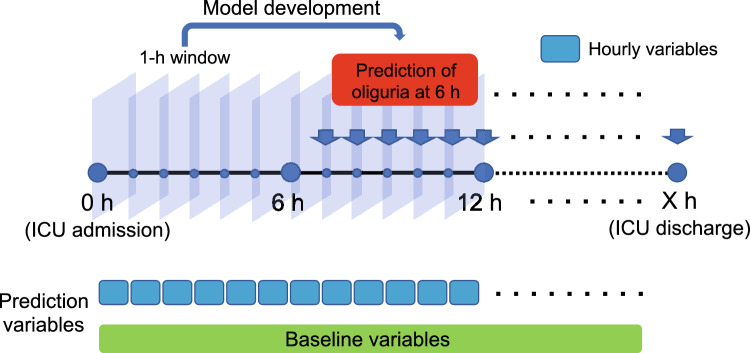
Schematic of the sequential machine-learning model for predicting oliguria. The sequential machine-learning model to predict oliguria occurrence at 6 h was developed using hourly variables and baseline information obtained during the ICU stay. The input features were updated to include a 1-h window of the previous values for the physiological parameters, blood tests, and medications. In terms of laboratory tests, the most recent variables were used. *ICU* intensive care unit.

**Figure 2:**
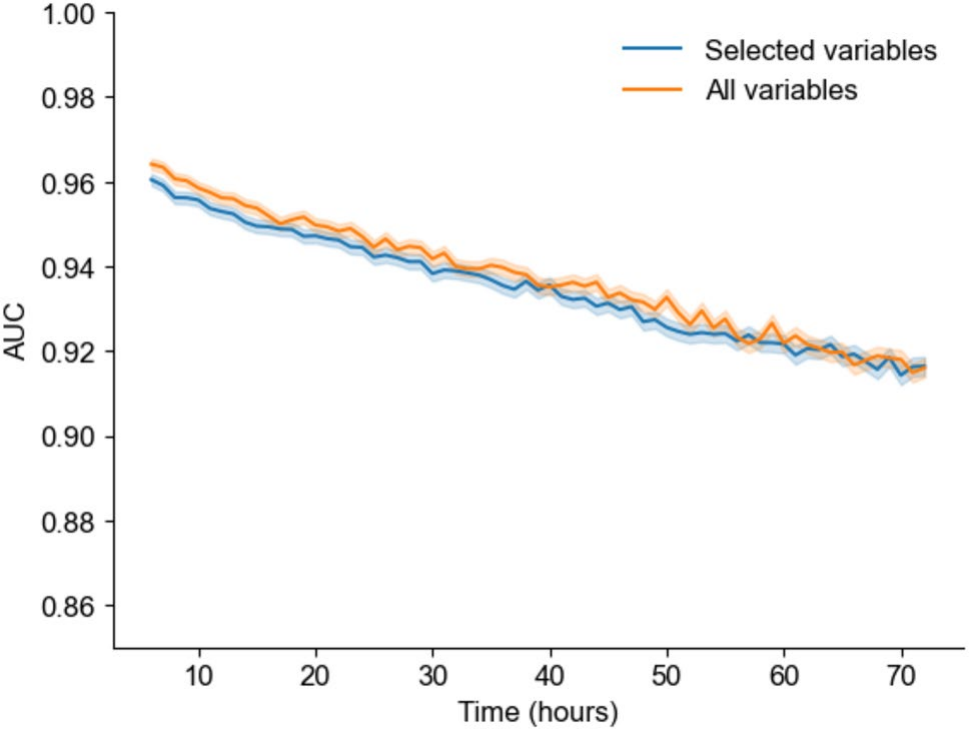
Sequential accuracy of the machine-learning algorithm for predicting oliguria in the intensive care unit. The line plot depicts the sequential changes in the AUC with the given time for the prediction algorithm for the selected variables (blue) and all variables (yellow). Additionally, the 95% confidence intervals of AUC are shown. *AUC* area under the curve.

We used hourly variables and baseline information to develop a sequential machine-learning model to predict oliguria (Fig. 1). From 1,018 variables, 28 variables were selected from a clinical perspective and included in the reduced dataset (Supplementary File: Table S1). Using the light-gradient boosting machine (LightGBM) classifier, we compared the accuracies of the models using all selected variables (Fig. 2). Although the area under the curve (AUC) values for predicting oliguria were comparable between the two methods, the computation time was much longer for the 1,018-variable dataset (56.3 s) than for the 28-variable dataset (7.5 s). The top 50 important variables in the 1,018-variable dataset overlapped with approximately 40% of the selected variables (Supplementary File: Fig. S2). To improve the efficiency and comparative accuracy of the algorithm, we used the 28-variable dataset for further analysis.

### Prediction of oliguria

**Figure 3:**
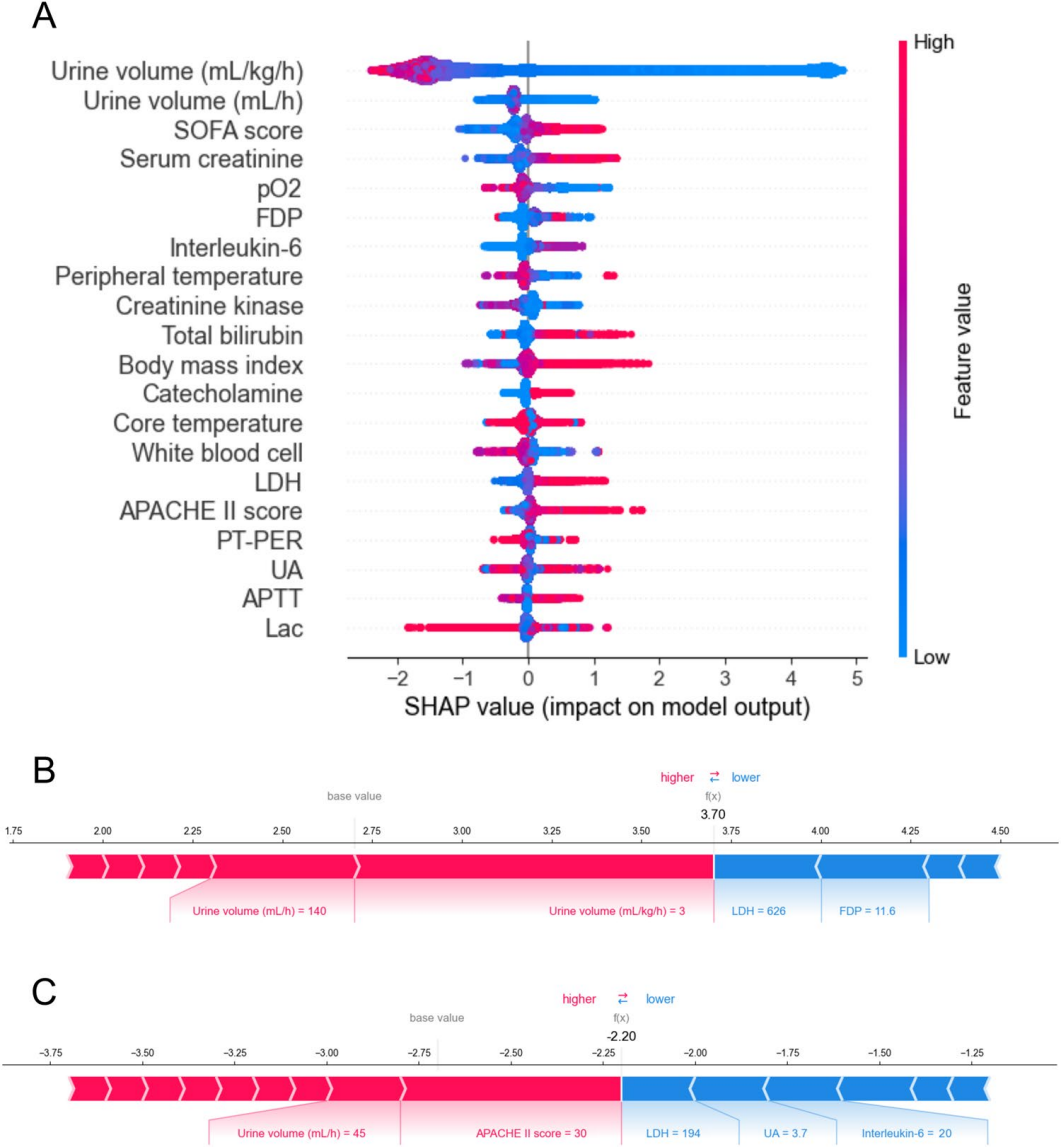
SHAP values of the machine-learning algorithm for predicting oliguria in the intensive care unit. (**A**) The effect of the features on the output of the model was manifested as the SHAP value. The features are placed in a descending order of importance. The correlation between the feature value and SHAP value reveals the beneficial or adverse impact of the predictor. The magnitude of the value is represented by the red (high) or blue (low) graphs. (**B**), (**C**) SHAP individual force plots illustrate the responsible features at scale with a color bar that encompasses the feature contribution to the onset of oliguria in individual cases. Two examples of patients presenting with a high probability of non-oliguria (**B**) or oliguria (**C**) are shown. *SHAP* Shapley additive explanations, *SOFA* sequential organ failure assessment,* pO*_2_ oxygen partial pressure, *FDP* fibrinogen degradation products, *LDH* lactate dehydrogenase, *APACHE* acute physiology and chronic health evaluation, *PT-PER* prothrombin time; *UA* uremic acid, *APTT* activated partial thromboplastin time, *Lac* lactate.

The AUC of the model to predict oliguria at 6 h using the selected variables was 0.964 (with a 95% confidence interval [CI] of 0.963–0.965). To verify the accuracy of the model, a fivefold cross-validation was implemented with an AUC of 0.920 (95% CI 0.918–0.922). The following Shapley additive explanation (SHAP) values are important variables for predicting oliguria at 6 h: the urine values, sequential organ failure assessment (SOFA) score, serum creatinine, oxygen partial pressure (pO_2_), fibrinogen/fibrin degradation products (FDP), interleukin (IL)-6, peripheral temperature, creatinine kinase, and total bilirubin (Fig. 3A). The SHAP individual force plots (Fig. 3B,C) show the SHAP values for two patients with and without oliguria. In the first patient, a greater urine volume decreased the probability of oliguria occurrence, whereas the elevation of lactate dehydrogenase (LDH) and FDP increased the probability of oliguria (Fig. 3B). Consequently, the model predicted a lower probability of oliguria occurrence at 6 h. In the second patient, a lower urine volume and higher acute physiology and chronic health evaluation (APACHE) II increased the probability of oliguria occurrence, whereas normal levels of LDH, uric acid (UA), and IL-6 decreased the probability of oliguria. Based on this information, the model predicted an increased risk of oliguria at 6 h (Fig. 3C). The prediction model for the onset of oliguria at 72 h still showed high accuracy (AUC of 0.916 [95% CI 0.914–0.918]). The important variables for predicting oliguria at 72 h based on the SHAP values overlapped with those at 6 h except for urea nitrogen and platelets (Supplementary File: Fig. S3). After analyzing the same dataset using the different computer setting as a sensitivity analysis, we obtained the same results as the primary analysis.

### Subgroup analyses

**Figure 4:**
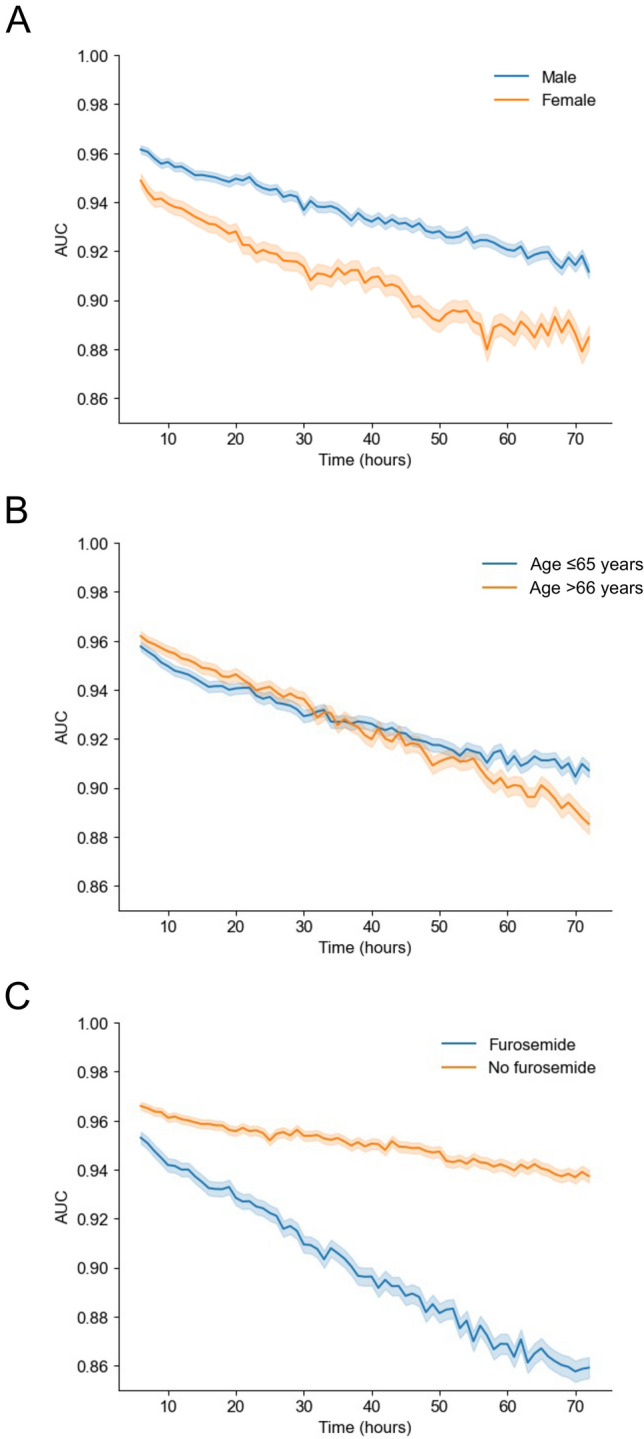
Subgroup analyses of prediction algorithms for the onset of oliguria in the intensive care unit. The line plot depicts the sequential changes in the area under the curve (AUC) for a given time for the prediction algorithm in the categories of (**A**) sex, (**B**) age, and (**C**) furosemide administration. Differences in the curves of the AUC plots between the two groups were examined using the mean absolute error (MAE) calculated from the averages of the summed absolute values of the differences in AUCs from 6 to 72 h. The MAE values for sex, age, and furosemide administration were 0.026, 0.006, and 0.050, respectively. Moreover, the 95% confidence intervals of the AUC are shown.

Next, we analyzed the accuracy of the models in predicting the onset of oliguria at 6 h according to sex, age (≤ 65 and > 66 years), and furosemide administration (Fig. 4A). In this context, the male group was more accurate (AUC = 0.965 [95% CI 0.964–0.967]) than the female group (AUC = 0.946 [95% CI 0.943–0.949]; mean absolute error [MAE] = 0.026). In the age comparison, there was a relatively small difference (MAE = 0.006) between the group younger than 65 years old (AUC = 0.958 [95% CI, 0.958–0.959]) and the group older than 66 years (AUC = 0.962 [95% CI, 0.960–0.964]) (Fig. 4B). Finally, the accuracy of the prediction model was higher for the non-furosemide group (AUC = 0.966 [95% CI 0.964–0.967]) than for the furosemide group (AUC = 0.953 [95% CI 0.951–0.955]), with a greater difference at a later prediction time point (MAE = 0.050) (Fig. 4C).

## Discussion

This study developed a machine-learning model with a high AUC (> 0.96) for predicting the onset of oliguria at 6 h in critically ill patients. Among the 28 clinically relevant variables used for the prediction model, urine values, the SOFA score, serum creatinine, pO_2_, FDP, IL-6, and peripheral temperature were listed as important variables.

Over the past decade, machine learning algorithms for predicting oliguria in critically ill patients have been underexplored, despite oliguria being one of the key components of AKI that leads to increased mortality in such patients^1,16–18^. Although several studies have repeatedly verified the precision of a machine-learning model to predict AKI in critically ill patients with an AUC range of 0.74 to 0.93^19–26^, only one study reported a machine-learning approach to predict oliguria for the next 4 h in patients with sepsis after fluid resuscitation using 47 clinical values, with an AUC of 0.86^15^. Our machine-learning model exhibited a high AUC (> 0.90) for predicting oliguria in critically ill patients between 6 and 72 h. The high accuracy of our model and its capability to predict oliguria over longer periods and fact that the AUC remained the same even after reducing the variables in the model development are a testament to the novel contributions of this study. This high accuracy may be attributed to the large sample size of > 10,000 patients, resulting in abundant training data. In addition, our method of predicting the onset of oliguria from an arbitrary time may have improved the accuracy by increasing the number of training datasets. Although we built the model based on 28 clinically relevant variables, its high overlap with the top-listed variables in the 1,018-value dataset would support the plausibility of using the selected variables for the prediction model. Because oliguria could identify more patients with AKI earlier than serum creatinine alone and is associated with poor outcomes in critically ill patients, our model would be useful for the early detection of patients with AKI and for improving the prognosis of the population through better management and early intervention^10,11,27,28^.

The SHAP analysis identified urine values, the SOFA score, serum creatinine, pO_2_, FDP, IL-6, and peripheral temperature as significant predictors of oliguria. Previous studies using SHAP values identified significant factors that increased the risk of ICU-acquired AKI, including a higher body mass index on admission, the presence of chronic kidney disease, congestive heart failure, coagulation and bleeding disorders, and cardiac arrhythmias, in addition to renal function represented by an elevation in blood urea nitrogen or serum creatinine, but not IL-6^19,24^. Because IL-6 is one of the representative cytokines induced by overwhelming systemic inflammation, several studies have reported the significance of blood IL-6 levels for early detection of multiple organ dysfunction^29–31^. The high weight of IL-6 in the development of the predictive model verified the essential role of this cytokine in the progression of organ dysfunction. IL-6 gained wide recognition in the cytokine storm associated with COVID-19 and can now be measured using rapid kits using immunoassays. Moreover, it has emergency approval in the United States^32^. In a previous study investigating the association between AKI and oliguria in critically ill patients, the SOFA score was higher in patients with oliguria than in those without oliguria in the AKI cohort^33^. In addition, a multicenter study identified the APACHE II score as a predictor in patients with acute renal failure, requiring dialysis^34^. These findings support our hypothesis that severity scores play an important role in the early detection of oliguria^35^. In summary, these important variables can provide insights into the complex pathophysiology of AKI.

In the subgroup analyses, the accuracy of the model in the female and furosemide groups decreased over time compared with that in the other groups, with relatively high accuracy (AUC = 0.86) even at 72 h after the time of observation. A previous study showed that males developed AKI in the ICU more frequently than females due to the protective function of estrogen, which includes proliferative and anti-apoptotic effects on proximal tubular cells. Therefore, physiological differences between the sexes may have affected the accuracy of the model^36,37^. Additionally, the administration of furosemide, which is arbitrarily timed by a physician, has a direct effect on urine output; therefore, it is conceivable that this subjective intervention could bias the accuracy of the model.

However, this study had the following limitations. First, this retrospective single-center study model may cause uncertainty when applied to different settings although we confirmed the accuracy of the cross-validation methods. Therefore, a prospective study in a different setting is required for future clinical applications. Second, we reduced the number of variables in this study from a clinical perspective; however, we may have missed important variables that affect prediction accuracy. Third, the SHAP analysis included both plausible and uninterpretable variables to predict outcomes. A mechanistic study investigating the significance of these uninterpretable values may reveal their role in the onset of oliguria and AKI. Future research should include an external validation through multicenter studies to verify the prediction accuracy. A prospective investigation is also warranted to evaluate the effects of model application on clinical outcomes.

In conclusion, this study demonstrated that a machine-learning model could predict the onset of oliguria in critically ill patients with high accuracy. Future investigations can focus on validating the accuracy of the prediction model for the early detection of AKI in patients.

## Methods

### Subjects

This retrospective cohort study used the electronic health record data of consecutive patients admitted to the ICU at Chiba University Hospital, Japan, from November 2010 to March 2019. The annual number of patients admitted to the 22-bed surgical/medical ICU ranged from 1,541 to 1,832. We excluded patients on maintenance dialysis and those without a documented body weight. This study was approved by the Ethical Review Board of Chiba University Graduate School of Medicine (approval number: 3380) in accordance with the Declaration of Helsinki. The Ethical Review Board of Chiba University Graduate School of Medicine waived the requirement for written informed consent in accordance with the Ethical Guidelines for Medical and Health Research Involving Human Subjects in Japan.

### Definition of oliguria and AKI

We defined oliguria as urine output of less than 0.5 mL/kg/h according to the Kidney Disease: Improving Global Outcomes stage I criteria. AKI was diagnosed based on an increase in serum creatinine level of at least 0.3 mg/dL from the baseline or oliguria^38^.

### Data collection

Patient records from the ICU data system contained 1,031 input variables, including (A) physiological measurements acquired every minute (heart rate, blood pressure, respiratory rate, peripheral oxygen saturation, and body temperature), (B) blood tests (complete blood count, biochemistry, coagulation, and blood gas analysis), (C) name and dosage of medications, (D) type and amount of blood transfusion, (E) patient observation record, and (F) patient care record. The minute-by-minute time-series tables were aggregated into hourly time-series tables. In the process of aggregating the tables, the median value was used for physiological measurements and the blood test values were obtained from the most recent test. For patient excretion values, urine and stool volumes were calculated as one-hour sums. The following six calculated variables were added to the dataset: hourly intake, hourly output, hourly total balance, hourly urine volume (mL/kg), oliguria (urine volume of less than 0.5 mL/kg/h), and oliguria for six consecutive hours. A total of 222 background information variables, including age, sex, and admission diagnosis, were also added to the dataset. Consequently, the dataset contained 1,127 variables. We treated the missing values as a separate group or excluded them from the analysis. To remove potential collinearity values, we performed a multicollinearity test and analyzed the data without these values.

### Machine learning algorithms and statistical analyses

The dataset was randomly divided: 80% for training and 20% for testing. We developed a sequential machine-learning model to predict oliguria at any given time during the ICU stay using hourly variables and baseline information (Fig. 1). For the values that were not continuously obtained, we used the most recent ones for the model development. The input variables were updated to encompass a 1-h window of the preceding values for the physiological measurements, blood tests, and medications. The primary and secondary outcome variables were oliguria at 6 and 72 h after an arbitrary time point from ICU admission to discharge, respectively. Accordingly, we used variables recorded until 6 or 72 h before ICU discharge corresponding to each outcome variable. The outcome variable was not incorporated as a predictor in the final model. After constructing the algorithm with the training data, the model predictions were validated using the test data. We validated the model performance with a fivefold cross validation. To ensure that the estimated model probabilities aligned with the actual probabilities of oliguria occurrence, we plotted the calibration curve of the model. The curve indicated that our model was well calibrated (Supplementary File 1: Fig. S4).

We selected four representative machine learning classifiers: LightGBM, category boosting (CatBoost), random forest, and extreme gradient boosting (XGboost). Before developing the prediction model, we compared the computational performances and model accuracies using the four classifiers (Supplementary File 1: Table S2). To develop the machine learning algorithm, we used a cloud computer (Google Collaboratory memory 25 GB) to evaluate the accuracy of the model. The AUC values based on the receiver operating calibrating curves, sensitivity, specificity, and F1 score were calculated. Among the machine learning classifiers, LightGBM showed the best computation speed and AUC and the second-best F1 score with a marginal difference from XGboost (XGboost 0.899, LightGBM 0.896). Based on these results, we decided to use LightGBM for the analysis in this study. After developing a prediction model with all the variables, we reduced the number of variables for prediction by selecting clinically relevant variables (Supplementary File: Table S2). Subsequently, we compared the performances of the LightGBM model using the selected variables and all the variables. As a sensitivity analysis, we re-analyzed the data using a different computer environment, Amazon Web Service Sagemaker. The computer settings included the following: image: Data Science 3.0, kernel: python 3, and instance type: ml.t3.medium (memory 64 GB).

To evaluate the important variables contributing to building the prediction model, we used the SHAP value. The SHAP value indicates the impact of each feature on the model output, with higher interpretability in machine learning models. We expressed the SHAP value as an absolute number with a positive or negative association between the variable and outcome. SHAP individual force plots showed several features at scale with a color bar that indicated the feature contribution to the onset of oliguria in individual instances, enhancing the interpretability regarding the connection between traits and the occurrence of oliguria. For the subgroup analyses, we compared the accuracies of the models in predicting oliguria based on sex, age (≤ 65 or > 66 years), and furosemide administration. To quantify the differences in the AUC plots of the two groups, the absolute values of the differences in the AUCs of each group from 6 to 72 h were summed and averaged to obtain the MAE.

Data were expressed as medians with interquartile ranges for continuous values and as absolute numbers and percentages for categorical values. A *P* value < 0.05 was considered as statistically significant. The main Python packages used in the analysis to create the machine learning algorithms were Python 3.10.11, pandas 1.5.3, numpy 1.22.4, matplotlib 3.7.1, scikit-learn 1.2.2, XGboost 1.7.2, lightgbm 2.2.3, catboost 1.1.1, and shap 0.41.0.

### Supplementary Information

Supplementary Information.

## Data Availability

The datasets used and analyzed in this study will be available from the corresponding author upon reasonable request.
