# Supplementary material for: Machine-learning model for predicting oliguria in critically ill patients
Source: Sci Rep. 2024 Jan 11;14:1054. doi: 10.1038/s41598-024-51476-y (PMC10784288; doi:10.1038/s41598-024-51476-y)
Supplement: Supplementary file 1 — Supplementary Information. [file 41598_2024_51476_MOESM1_ESM.pdf]

Machine-learning model for predicting oliguria in critically ill patients

Yasuo Yamao[1], Takehiko Oami[1], Jun Yamabe[2], Nozomi Takahashi[1], Taka-aki Nakada[1]

Affiliations:

1. Department of Emergency and Critical Care Medicine, Chiba University Graduate School of Medicine, Japan
2. Smart119 Inc., Japan

Corresponding author

Taka-aki Nakada

Department of Emergency and Critical Care Medicine, Chiba University Graduate School of Medicine, 1-8-1 Inohana, Chuo, Chiba, 260-8677, Japan

Phone: +81-43-226-2372, Fax: +81-43-226-2371

Email: taka.nakada@nifty.com

Supplementary information files

Table S1. Selected variables for developing a machine-learning algorithm

Table S2. Accuracies and computational times of machine-learning classifiers

Figure S1. Flowchart for the enrollment of patients in the study

Figure S2. Top 50 important variables in the 1,018-value dataset for predicting oliguria at 6 h

Figure S3. SHAP values of the machine learning algorithm for predicting oliguria at 72 h in the ICU

Figure S4. Calibration curve of the machine learning algorithm for predicting oliguria

Table S1. Selected variables for machine-learning algorithm development

Data source	Variables
ICU admission	Age*, Sex, SOFA score, APACHE II score
Physiologic measurements	Body mass index, Systolic blood pressure*, Mean blood pressure*, Diastolic blood pressure*, Heart rate*, Respiratory rate*, oxygen saturation*, Central venous pressure, Core temperature, Axillary temperature
Blood tests	AST, ALT, LDH, CPK, Total protein*, Albumin*, Uremic acid, Urea nitrogen, Creatinine, Total bilirubin, CRP, Interleukin-6, White blood cell, Hemoglobin*, Platelet, PT-INR, APTT, FDP, pH*, pCO_2*, pO_2, HCO_3^-*, Base excess*, Sodium*, Potassium*, Chloride*, Calcium*, Lactate
Observational record	Urine volume (mL/h)
Calculated data	Urine volume (mL/kg/h)<br>Total amount of input*<br>Total amount of output*<br>Water balance*<br>Administration of cardiovascular agents<br>Administration of diuretics<br>Administration of transfusion

SOFA: sequential organ failure assessment; APACHE: acute physiology and chronic health evaluation; AST: aspartate aminotransferase; ALT: alanine transaminase; LDH: lactate dehydrogenase; CPK: creatinine phosphokinase; CRP: C-reactive protein; PT-INR: prothrombin time-international normalized ratio; APTT: activated partial thromboplastin time; FDP: fibrinogen degradation product

* Potential collinearity values are removed from the selected variables for developing the machine-learning algorithms.

Table S2. Accuracies and computational times of machine-learning classifiers

Classifier	Computation time (s)	AUC	F1 score
LightGBM	83	0.984	0.896
CatBoost	454	0.982	0.877
RandomForest	622	0.976	0.842
XGboost	1,480	0.984	0.898

AUC: area under the receiver operating characteristic curve; GBM: gradient boosting machine; CatBoost: category boosting; XGboost: extreme gradient boosting

Figure S1. Flowchart for the enrollment of the patients in the study

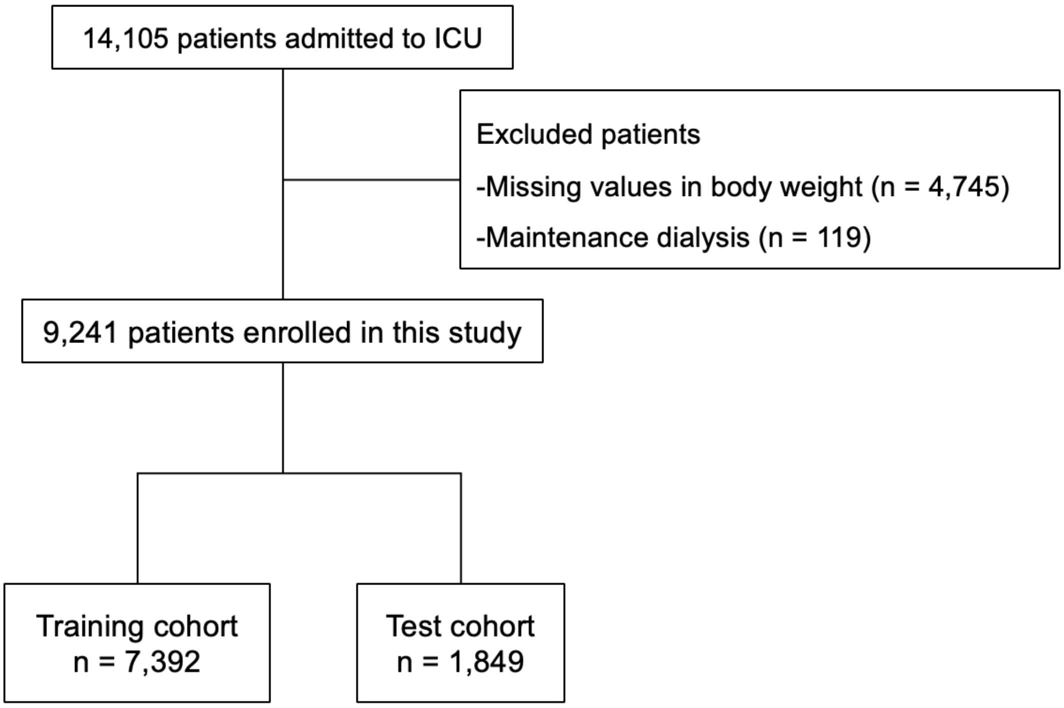

ICU: intensive care unit

Figure S2. Top 50 important variables in the 1,018-value dataset for predicting oliguria at 6 h

Rank	Item	Importance
1	Urine volume (mL/kg/hr)	243
2	Serum creatinine on admission	89
3	SOFA score	65
4	Activated clotting time	55
5	Weight	50
6	Serum creatinine	49
7	Lactate	48
8	Alkaline phosphatase on admission	38
8	Total amount of output	38
10	Uremic acid	31
10	Heart rate	31
12	Non-invasive systolic blood pressure	30
12	LDH on admission	30
14	Lymphocyte on admission	29
15	Interleukin-6	28
15	Age	28
15	Heart rate on admission	28
15	Height	28
19	Core temperature	27
19	Dose of noradrenaline	27
19	Non-invasive systolic blood pressure	27
19	Urea nitrogen on admission	27
23	Dose of furosemide	26
24	Platelet	25
24	MCHC on admission	25
24	Glucose on admission	25
24	Prothrombin time on admission	25
28	Calcium	24
28	Peripheral temperature	24
28	Non-invasive diastolic blood pressure	24
31	FDP	23
31	Respiratory rate	23
33	AST	22
33	Pulse rate on admission	22
33	Oxyhemoglobin on admission	22
36	LDH	21
36	Prothrombin time activity percentage	21
36	White blood cell	21
39	Alkaline phosphatase	20
39	Admission to the Department of Neurosurgery	20
41	Gamma-glutamyl transpeptidase	19
41	Lymphocyte	19
41	Deoxyhemoglobin on admission	19
44	Activated partial thromboplastin time	18
44	Glutamic acid	18
44	Mean corpuscular volume on admission	18
44	Enteral route	18
48	β-D glucan	17
48	SpO_2	17
48	Creatine phosphokinase on admission	17
48	Estimated mortality by APACHE2	17

The top 50 important variables in the 1,018-value dataset for predicting oliguria at 6 h are listed in descending order of importance. Rows with a yellow background overlap the selected clinically relevant variables.

SOFA: sequential organ failure assessment; LDH: lactate dehydrogenase; MCHC: mean corpuscular hemoglobin concentration; FDP: fibrinogen degradation product; AST: aspartate aminotransferase; SpO_2: oxygen saturation; APACHE: acute physiology and chronic health evaluation

Figure S3. SHAP values of the machine-learning algorithm for predicting oliguria at 72 h in the intensive care unit

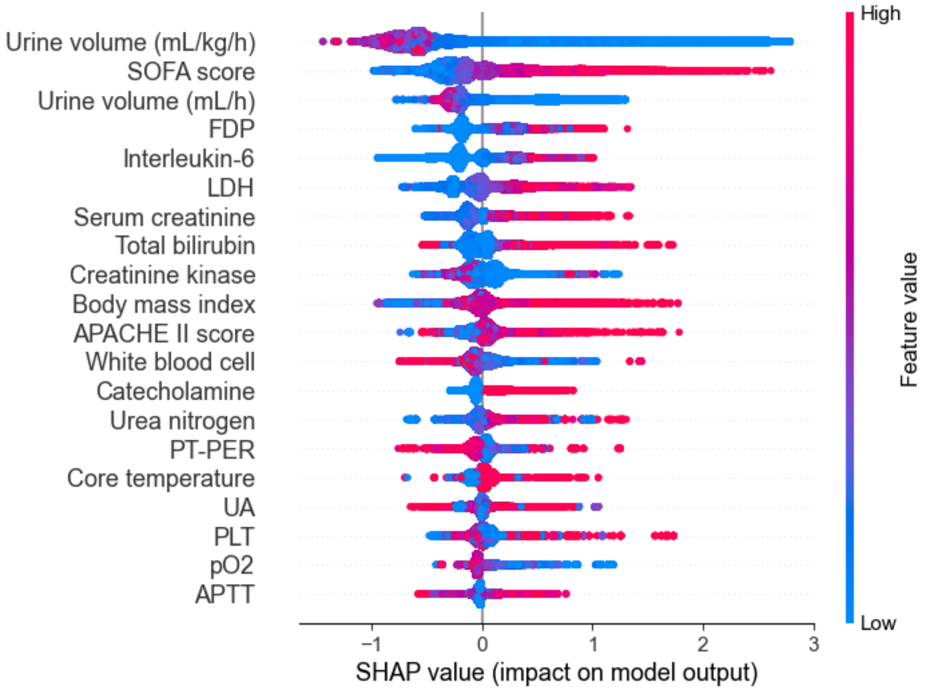

The effect of a feature on the output of the model was manifested as the SHAP value. The features are placed in descending order of importance. The correlation between the feature value and SHAP value reveals the beneficial or adverse impact of the predictor. The magnitude of the value is represented by the red (high) or blue (low) graphs.

SHAP: Shapley additive explanations; SOFA: sequential organ failure assessment; FDP: fibrinogen degradation product; LDH: lactate dehydrogenase; APACHE: acute physiology and chronic health evaluation; PT: prothrombin time; UA: uremic acid; PLT: platelet; pO_2: oxygen partial pressure; APTT: activated partial thromboplastin time

Figure S4. Calibration curve of the machine learning algorithm for predicting oliguria

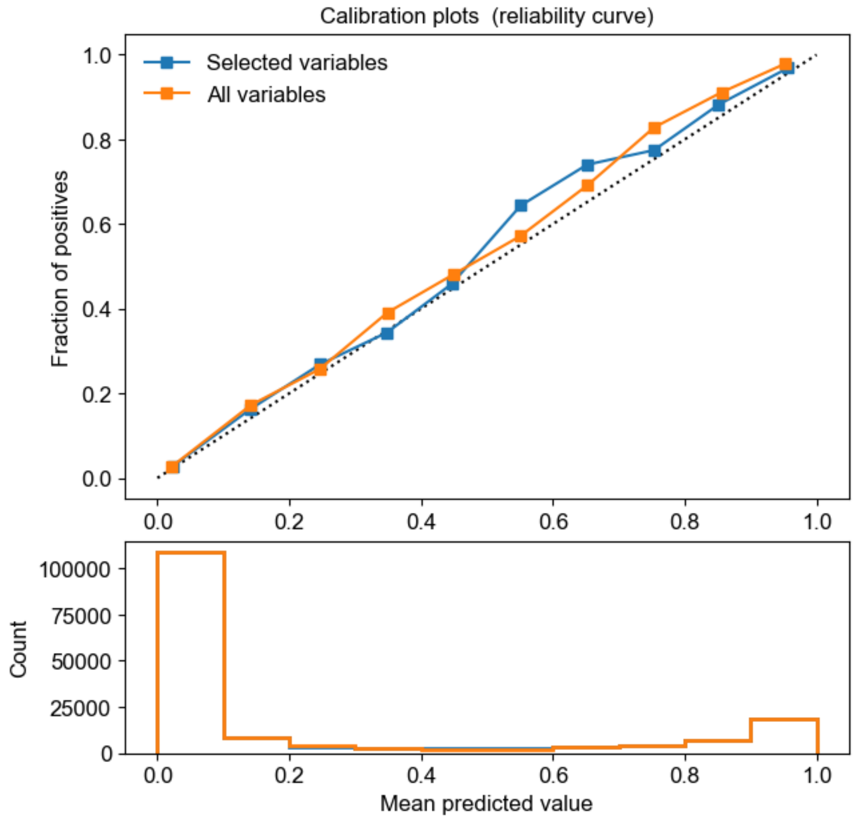

The calibration plots illustrate the precision probabilities of LightGBM for predicting oliguria using all the variables or selected variables. The dotted line indicates perfect calibration, equal probability between the model and actual occurrence. The lower panel shows the number of cases in each predicted probability bin.
